# MCMC-ODPR: Primer design optimization using Markov Chain Monte Carlo sampling

**DOI:** 10.1186/1471-2105-13-287

**Published:** 2012-11-05

**Authors:** James L Kitchen, Jonathan D Moore, Sarah A Palmer, Robin G Allaby

**Affiliations:** 1 School of Life Sciences, University of Warwick, Gibbet Hill, Coventry, CV4 7AL, UK; 2 Warwick Systems Biology, University of Warwick, Coventry, CV4 7AL, UK

## Abstract

**Background:**

Next generation sequencing technologies often require numerous primer designs that require good target coverage that can be financially costly. We aimed to develop a system that would implement primer reuse to design degenerate primers that could be designed around SNPs, thus find the fewest necessary primers and the lowest cost whilst maintaining an acceptable coverage and provide a cost effective solution. We have implemented Metropolis-Hastings Markov Chain Monte Carlo for optimizing primer reuse. We call it the Markov Chain Monte Carlo Optimized Degenerate Primer Reuse (MCMC-ODPR) algorithm.

**Results:**

After repeating the program 1020 times to assess the variance, an average of 17.14% fewer primers were found to be necessary using MCMC-ODPR for an equivalent coverage without implementing primer reuse. The algorithm was able to reuse primers up to five times. We compared MCMC-ODPR with single sequence primer design programs Primer3 and Primer-BLAST and achieved a lower primer cost per amplicon base covered of 0.21 and 0.19 and 0.18 primer nucleotides on three separate gene sequences, respectively. With multiple sequences, MCMC-ODPR achieved a lower cost per base covered of 0.19 than programs BatchPrimer3 and PAMPS, which achieved 0.25 and 0.64 primer nucleotides, respectively.

**Conclusions:**

MCMC-ODPR is a useful tool for designing primers at various melting temperatures at good target coverage. By combining degeneracy with optimal primer reuse the user may increase coverage of sequences amplified by the designed primers at significantly lower costs. Our analyses showed that overall MCMC-ODPR outperformed the other primer-design programs in our study in terms of cost per covered base.

## Background

Next generation sequencing technology has led to the frequent application of deep sequencing projects, and the use of systems that require a large number of oligonuceotide primers for PCR. The design of a high number of primers is a challenge logistically both in terms of achieving good coverage of target regions and in terms of cost. Although there are a number of primer design programs available, utilizing them for high throughput design can be difficult and financially costly. We aimed to produce a system in which a large number of primers could be designed cost effectively by using the fewest necessary primers, hence the lowest cost, at multiple priming sites where possible whilst maintaining an acceptable level of coverage, and avoiding degeneracy in amplicon targets which overlap in the same regions.

In designing our program we compared our approaches and performance with several other available programs; Primer3 [1], Batchprimer3 [2], Primer-BLAST [3] and PAMPS [4]. The core algorithm for the first three of these programs is that of Primer3. The Primer3 algorithm takes into account the primer size, melting temperature (T_m_), GC content, and concentration of monovalent and divalent cations within the PCR reaction mixture, a selection of salt correction formulae and different parameters for simulating the thermodynamics of primer hybridization. Potential primers are then checked by using a mispriming repeat library from the human, rodent or *Drosophila* genomes, allowing interspersed repeats or other sequence regions to be avoided as primer annealing locations. Primer-BLAST utilizes the Primer3 algorithm and the BLAST local alignment search tool [5] to ensure only unique primer pairs are selected, thus preventing primers becoming designed around undesired targets such as introns. These two programs output a range of primer pair possibilities for single DNA sequences, but they are not designed for high throughput primer design. The need for high throughput primer design was recognized by You et al. [2], who produced BatchPrimer3 in which multiple sequences can be input for primer selection, but only one primer pair per input sequence is produced.

Minimizing the cost of a primer design can be achieved by (i) designing degenerate primers able to anneal to a number of related target sequences and (ii) implementing primer reuse utilizing primers that bind to conserved loci that are repeated. Although degeneracy allows for amplification of greater numbers of related sequences, the more degenerate primers are the less specific amplicons will be. Therefore achieving an optimal degree of degeneracy is important to obtaining a suitable trade-off between the number of related sequences amplified and the specificity of these amplicons. A number of variants exist for tackling this problem and achieving a good trade-off for the specificity and sensitivity. The Maximum Coverage Degenerate Primer Design (MCDPD) approach as used in HYDEN [6] tries to identify a primer of length *l* and a maximum degeneracy *d*_*max*_ that covers a maximum number of sequences, each of length *l*. The Minimum Degeneracy Degenerate Primer Design (MDDPD) attempts to find a primer of length *l* and a minimum degeneracy *d*_*min*_ that can cover all input sequences with a length equal to or greater than *l*. The Minimum Primers Degenerate Primer Design (MPDPD) attempts to find the fewest number of primers of length *l* and a maximum degeneracy of *d*_*max*_ for a set of sequences, so that each sequence is covered by at least one primer. Whereas this approach has the constraint that all input sequences have the same length as the primers and may be inadequate in practice, the Multiple Degenerate Primer Design (MDPD) allows the input sequences to have different lengths of greater than *l*_*min*_ and attempts to identify primers of length at least *l*_*min*_ and degeneracy *d*_*max*_, allowing each sequence to be covered by at least one primer. This was the approach taken by MIPS [7] and PAMPS [4]. PAMPS is a heuristic, high throughput algorithm which designs degenerate primers through a process of consecutive *ad hoc* pairwise alignments [4]. This program has been shown to outperform other degenerate primer design systems such as HYDEN and MIPS in terms of computational time.

Algorithms for implementing primer reuse have also been developed: Doi and Imai have described a heuristic algorithm for greedy primer design within multiplex PCR, which attempts to minimize the cost of primers required for multiplex PCR and SNP genotyping [8]. MuPlex is another heuristic algorithm designed for multiplex PCR, which uses a graph based approach to assign the largest number of non-conflicting primers into the fewest ‘cliques’ that can be assigned to multiplex PCR tubes [9]. Lui and Carson have utilized a simulated annealing optimization to maximize primer reuse, which exhaustively searches primer space and aims to converge upon the optimal cost solution [10]. Despite individual cost benefits from either optimization of primer reuse, or automated design of degenerate primers, combining the two techniques is likely to offer additional cost advantages.

Optimizing primer design to make use of degeneracy and multiplexing has been referred to as the Multiple Degenerate Primer Selection Problem (MDPSP), and variants have been shown to be NP-complete. Previous approaches to MDPSP, such as those undertaken by Balla et al [11] have shown that primer coverage and cost can be improved through approximate (heuristic) greedy algorithms. Jabado *et al*. provide a heuristic algorithm for degeneracy, Greene SCPrimer [12]. In this method phylogenetic trees are constructed from multiple sequence alignments to identify candidate primers, which are used by a greedy set covering problem (SCP) solving algorithm to determine the minimum set of degenerate primers that may amplify all members of the alignment, so combining degeneracy with primer reuse. Although heuristic approaches generally outperform global optimizations in computation time, the reverse can potentially be true in quality of output. Given that optimization of large multiplexed primer design is not generally time-critical, a global optimization approach seems appropriate.

In order to improve on greedy approaches to MDPSP we present here an algorithm that takes a Markov Chain Monte Carlo (MCMC) approach, which allows sampling through primer parameter space using a probability distribution of acceptance of iterative primer designs. Primers are weighted according to their degree of reuse provided their degeneracy is kept below a user-defined threshold. We have implemented a Metropolis-Hastings algorithm, in which new proposals (e.g. the cost of a primer design) are accepted if they provide a more optimal solution to the current proposal, with the system tending to revert probabilistically, to the current state if the new proposal is more costly. We call the algorithm the Markov Chain Monte Carlo Optimized Degenerate Primer Reuse (MCMC-ODPR) algorithm. We show that the MCMC-ODPR program outperforms Primer3, Primer-BLAST, BatchPrimer3, and PAMPS in terms of cost and in terms of sequence coverage.

## Implementation

The main goal of MCMC-ODPR is to optimize the cost of a design by introducing degeneracy into primers and stochastically searching the cost landscape. The goal is to find the fewest necessary primers (lowest cost), whilst maintaining an acceptable coverage throughout all input target sequences. We define the cost as the number of nucleotides present in a set of primers identified in a sequence set and define the coverage as the number of base pairs within the sequences that will be amplified by the primers identified for the set. We summarize the two aforementioned statistics as cost per covered bases which is defined as cost / coverage. For a given set of target sequences *S*, and a set of primer design constraints *C*, there are typically either 0 or very many possible designs *D*, each constituting a set of overlapping primer pairs, which can potentially amplify *S* in a multiplexed PCR reaction. Typical design constraints include the melting temperature, T_m_; the maximum and minimum primer pair length, *L*_*max*_, and *L*_*min*_; and the maximal desired degeneracy, *d*_*max*_. We aim to optimize *D*|(*S*, *C*). The full list of constraints that the primers belonging to the optimized design must satisfy are listed below:

1. The GC content of the primers must be taken into account. Due to the stronger intermolecular forces between the guanine and cytosine nucleotides, T_m_ is highly dependent on this, which in turn has implications for how well primers hybridize to template sequences and for specificity of amplicons.

2. Primers should be minimally degenerate with a limited number of degenerate nucleotides per primer.

3. The forward and reverse pair of primers must not allow amplification of a target that exceeds the desired target length.

4. The primers should ideally be used multiple times, to keep the financial cost down. However, primer pairs should also be unique, otherwise the specificity of the targets will be reduced.

5. Primers should not be designed that will hybridize to the target between a forward and reverse primer of an already established amplicon, unless this will lower the cost of the overall design.

6. Primers should not be designed which are complementary to other primers, i.e. form dimers with other primers.

7. Primers should not be designed which are likely to self-hybridize and form hairpin structures.

For a primer *i* of length *l*_*i*_ we assume:

(1) $${\text{cost}}_{i}\;\alpha \;l_{i}$$

Therefore, for a primer set design *D*, targeting sequences *S* that contain *k* distinct primers we assume:

(2) $${\text{cost}}_{D}\;\alpha \sum_{i=1}^{k}l_{i}$$

We attempt to minimize cost_*D*_ by allowing degeneracy in *i* and by sampling possible designs within design constraints using an MCMC sampler. Greedy approaches to minimizing *cost*_*D*_ in a primer set design for multiple target sequences will result in a final *cost*_*D*_ which depends in part on which of the target sequences is chosen first, to begin the design, and on which position within each sequence the primer design is begun for that sequence. We sample from uniform prior distributions on both of these parameters. We also allow our sampler to vary the position of individual primers within the design, and the degeneracy of individual bases within each primer within the chosen degeneracy limit, both also with uniform priors. Our sampler allows each of these parameters to vary, initially preferring to sample from the earlier of the four parameters and increasingly preferring to sample from the latter.

### Program input

For ease of use inputs to MCMC-ODPR are specified in a simple text document, which is then read into the program, Table 1. The input data to the program are three sets of nucleotide sequences in FASTA format. The first FASTA file contains genomic background sequences, which could be the whole genome, a large EST or GSS set, and will be searched using the BLAST local alignment search tool to asses the redundancy of possible primers and the uniqueness of forward and reverse primer pairs. The two remaining files provide MCMC-ODPR with the sequences to amplify and with SNP marker information respectively. The ‘sequences to amplify’ file contains a representative gene sequences (consensus sequence) for each entry while the SNP marker information file contains the same sequences with all collated SNPs for that gene, known as the exceptions sequences. Depending on the availability of SNP information, not all consensus sequences need be present in the exceptions sequence file, however. A small program has been included in the MCMC-ODPR package, EXCEPTIONS_GENERATOR, which on taking a FASTA file of all variants of sequences of genes to be amplified, will automatically generate the consensus and exceptions files. In the absence of SNP information, no exceptions file is necessary.

**Table 1:** Input file parameters for MCMC-ODPR

**Input parameter**	**Default value**
Genome file for BLAST searching	hv_mRNA_PUT.fas
Max degeneracy (base pairs)	3
Minumum melting temperature (centigrade)	50
Maximum melting temperature (centigrade)	60
Minimum amplicon length (base pairs)	50
Maximum amplicon length (base pairs)	250
Initial overlap (base pairs)	0
Number of optimisations	10000
Maximum gap between sequences (base pairs)	10
Save interim optimizations?	0
Verbose output?	0
Cost tolerance	0
Output file name	mcmc_odpr_results.out
Restart from previous run?	0
Probability of removing redundant primer pairs	0
Proportion of iterations to be considered as `early'	0
Weight greedy methods according to optimization?	1
Remove non-reusable primers from initial design?	0
Proportion of failed weight check proposals to accept (heating)	0.2

The parameters that may be input by the user into the MCMC-ODPR parameter file are in the left-most column. If the user chooses not to enter one of these parameters, then the default value on the right-most column will be used.

### MCMC-ODPR primer preparation stages

MCMC-ODPR initially goes through three stages of valid primer enumeration. Firstly, it searches all input consensus sequences for all possible primers meeting design constraints, whilst iterating through the input range of melting temperatures. At the second stage primer-dimers are removed and the redundancy of primers and uniqueness of primer pairs is assessed. Primers that have the propensity to self hybridize are subsequently removed. Primers are weighted according to their redundancy, with more highly weighted primers being preferred later in the optimization. In the third stage, for all remaining accepted primers, MCMC-ODPR recursively generates all possible degenerate primers to the input target level of degeneracy and tests whether they match other primers in the data set, to reassess their redundancy. In order to restrict a lack of specificity in the targets of the designed primers, the degree of degeneracy and redundancy must be limited, however. In the case of redundancy a high cut-off value allowing large redundancy values could favor primers being designed from low complexity or microsatellite regions of the target genes. A cut-off of three S or W degenerate bases yielding 2^3^ potential priming sites for degeneracy and a maximum redundancy of 10 are the default values in MCMC-ODPR; these can be changed by the user. After these initial stages, the Metropolis Hastings MCMC optimization begins.

### MCMC-ODPR degenerate primer design

When designing degenerate primers, it is important to maintain a degree of specificity within the primers by limiting the number of degenerate nucleotides per primer, otherwise designed primers will likely hybridize to numerous loci on the target sequence; generating highly unspecific amplicons and reducing the scope for primer reuse and cost effectiveness. Furthermore, if primer T_m_ is constrained, the possible combinations of degenerate loci that are permitted within a design must also be constrained to avoid generation of degenerate primers with variant T_m_. For instance, a primer having one degenerate position allowing an A or a C (IUPAC code M) would generate two primers with different T_m_ values, which is inappropriate if primers are being designed for a specific T_m_. For our purposes, therefore, the degree of possible degeneracy per primer is limited with primers having lower degeneracy, yet higher redundancy being more highly weighted for each proposal. Degenerate oligonucleotides which are permitted in our primer designs are W (bases A and T) and S (bases G and C). Take P to be the set of all possible primers extracted from the candidate gene set. A degeneracy threshold α is implemented and represents the maximum proportion of degenerate nucleotides within the primer. MCMC-ODPR then iteratively generalizes through all possible degenerate variations of the primers within P, whilst not contravening α. This exhaustive set is then searched during the greedy MCMC algorithms described below.

### MCMC-ODPR Greedy primer selection method

The primer cost optimization itself consists of three different greedy proposal generating methods that are progressively applied more frequently in succession. The first covers all input gene sequences by randomly selecting genes progressively and then picks primers from a random seeded position within the sequence, building amplicon targets from that seed in a periodicity which matches a user defined size range by randomly selecting amplicon sizes within that range to look for subsequent primer positions. This process is repeated, with the subsequent primer set being selected if the global cost is lowered. In this process primer loci may be ‘swapped’, with more reusable primers being accepted over less reusable primers. The second method (per-gene refinement) repeats the seeding process for each gene individually having accepted the global primer arrangement from the first step. The final greedy method adjusts final primer positions by repositioning each primer randomly. Again, if each primer adjustment results in a cost reduction, the adjustment is accepted. To allow the algorithm to converge upon a solution, it is desirable to allow a transition from optimizations that greatly affect a design to more ‘fine tuning’ optimizations. Therefore these methods may be weighted according to how many iterations within the optimization have passed: with the first greedy method being weighted for within the first third of all iterations, the second within the second third of optimizations and the third within the last third. These methods are stochastically chosen, however, hence all three greedy methods can still be chosen randomly by the algorithm at any time, allowing primer set design space to be sampled in different ways.

### Proportion of accepted failed proposals and proportions of ‘hot’ chains

The primer cost sample space may be visualized as a landscape, with various local cost minima and the global minima. The Metropolis-Hastings algorithm takes a solution proposal and then assesses whether this current proposal is more optimal than previous proposals. If this is the case, the Markov chain is allowed to continue stepping through the landscape. Otherwise the algorithm reverts back to a previous state. An initial random cost, C is proposed. At each iteration the cost associated with the new proposal, C’ is compared to C, with δS = C’-C. Unless δS is negative, the proposal is accepted with a probability proportional to e^-δSH^; where the heating H is a user input variable (see Table 1). The heating variable is defined as the proportion of primers that have failed their redundancy/weight check proposals that may be accepted, allowing chains to escape local minima and allowing efficient convergence. A variable specifying the proportion of optimizations to consider as ‘young’ is also input to allow convergence to be estimated in mature chains, as an alternative to specifying a fixed number of iterations.

### Tuning parameters and convergence

The parameter INITIAL_OVERLAP allows the user to build ‘slack’ into the initial proposal, allowing for a more extensive exploration of the cost landscape before the proposed solution tends towards being trapped in a local optimum. If larger values of INITIAL_OVERLAP are used, the initial proposal becomes more expensive because more primer pairs are used to cover the target sequences, but by initially proposing a more expensive design there is more scope for the algorithm to explore the cost landscape by changing the positions of individual primer pairs, whilst still satisfying the maximum amplicon length constraint. The parameter PROB_REMOVE_REDUNDANT constrains the rate at which this initial slack is removed from the design in cases where two neighboring primer pairs have changed position to the extent that the pairs become nested.

Currently the algorithm only aims to optimize cost, and not coverage. Because of this, as the design begins to approach an optimum cost for a given coverage, it will then likely allow coverage to reduce in order to further minimize the cost by removing one or more primer pairs from the design, for instance, and leaving the corresponding sequence uncovered.

### MCMC-ODPR output

The output of MCMC-ODPR is a single text file, containing an enumeration of all primers designed at each input T_m_, the selected degenerate primers, the number of covered bases and the final cost in nucleotides.

## Results and discussion

### MCMC-ODPR overall performance

**Figure 1:**
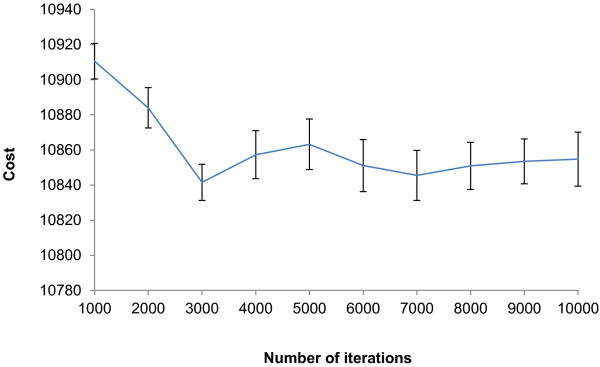
**Cost results of MCMC-ODPR over 10,000 optimizations.** The program was run 1000 times, with the first 100 being run to 1000 iterations, the second 100 run to 2000 iterations and so on until 10,000 iterations was reached. A dataset of 30 FASTA sequences was input, with no SNP processing specified and a melting temperature of 60 degrees centigrade only.

By observing the final cost results of the primer design every 1000 iterations and running MCMC-ODPR for a maximum of 10,000 iterations, we explored the effect of the number of MCMC iterations on the cost, Figure 1. Primers were designed with T_m_ = 60 degrees centigrade, and with an amplicon length between 50 and 250 bp. The algorithm was repeated 100 times and the final cost was recorded every 1000 iterations from 1000 – 10,000 iterations, to assess the variance in the convergence. A small set of 30 barley FASTA gene sequences were input into MCMC-ODPR due to the extra computational costs incurred by running the algorithm for these many runs (totaling at 1000 runs). Unlike heuristic approaches to primer design which calculates a set of primers, MCMC converges upon a stationary distribution giving the optimal primer set, through random chain mixing. We would therefore expect a degree of variance amongst repeated runs. Exact convergence can be difficult to measure with MCMC and a number of different diagnostic approaches have been suggested to allow this measurement to be made (for a review, see [13]). The samples drawn from a MCMC sampler will diverge from the prior distribution and approximate to the stationary distribution as the time taken tends to infinity, therefore the bias arising from these samples that is not representative of the prior distribution can be a measure of convergence to the stationary distribution. Furthermore, as the Markov chains converge there will be an increasing correlation between samples and the variance can therefore be another measure. These measures of convergence can be assessed graphically or quantitatively and some of the underlying diagnostic tools are applicable from one MCMC variant (such as Gibbs sampling or Metropolis-Hastings) to many. However, ultimately slow chain mixing can confound any diagnostic as the stationary distribution is always unknown. For our purposes, however, we were interested in validating our approach by observing a decrease in cost over time, therefore we observed the cost in the designed primers over a large number of iterations. An overall trend can be seen that as the number of iterations increases, the total cost decreases and the variance is small. However, the improvement in cost was slow after 10,000 iterations so we carried out all our analyses using this iteration number in this study.

To effectively assess the variance in the cost, coverage and the degree of redundancy achieved after 10,000 iterations, the MCMC-ODPR algorithm was run 1020 times. An average cost per covered base over the 1020 samples was 0.16 nucleotides with a standard error of 5.95x10^-5^ nucleotides. We analyzed the degree of redundancy that we obtained from the 1020 runs in Table 2. The vast majority of runs achieved 4x coverage, with the maximum degree of redundancy at 5x and the lowest redundancy at 2x. We also assessed the variance in the number of primers identified at each level of redundancy over the 1020 runs, Table 2. Standard errors were calculated for the number of primers at each degree of redundancy and it was clear that the variance was low. By multiplying the average number of identified primers in Table 2 by their degree of redundancy and then summing over these values, we arrive at a value of 654.84 primers required to obtain the same coverage when no redundancy is used. With redundancy, only 542.63 primers are needed (Table 2), a reduction of 17.14%.

**Table 2:** The variance in the number of primers obtained from 1–5x redundancy

**Redundancy**	**Frequency of redundancy value when maximum**	**Number of primers >0**	**Average number of primers identified**	**Standard error**
5x	14	14	0.01	0
4x	867	881	1.28	0.03
3x	130	1005	4.39	0.05
2x	9	1020	99.54	0.1
1x	0	1020	437.41	0.22

The algorithm was repeated 1020 times with a small subset of 30 sequences. The maximum achieved redundancy obtained by MCMC-ODPR in each run was recorded and when no primers were obtained at a certain level of redundancy, a zero was added as an observation, ensuring observations at all levels of redundancy summed to 1020. Column 2 gives the number of frequency of primers found at each level of redundancy throughout all 1020 runs when their numbers were greater than none. The average number of primers identified by the algorithm each run and the standard error is presented.

### MCMC-ODPR performance over a temperature range

**Figure 2:**
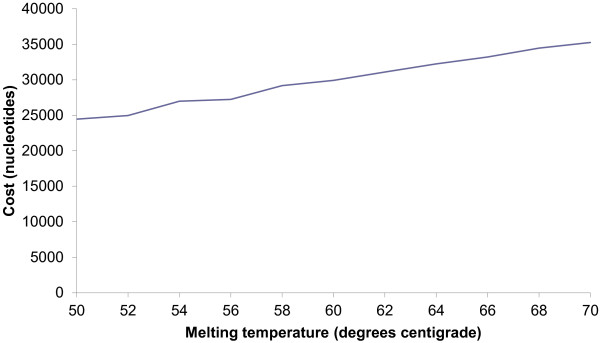
**Cost of the designed primers over a Tm range.** The dataset of 247 FASTA sequences were input into MCMC-ODPR with no SNP processing and 10,000 optimization iterations. The cost of the primers designed by MCMC-ODPR is plotted over a melting temperature range.

**Figure 3:**
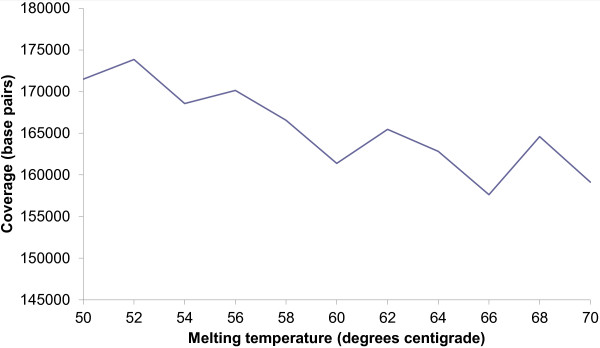
**Coverage of the designed primers over a Tm range.** The dataset of 247 FASTA sequences were input into MCMC-ODPR with no SNP processing and 10,000 optimization iterations. The percentage sequence coverage of target amplicons is plotted over a melting temperature range.

The MCMC-ODPR algorithm enumerates through the input T_m_ range and designs primer sets for each temperature, which allows inspection of the trade off between coverage and primer cost over the T_m_ range. A set of 247 FASTA sequences was input into MCMC-ODPR, and primers were recursively designed from a T_m_ range of 50 - 70°C. The optimization was run for 10,000 iterations at each temperature enumeration, with a target amplicon length range between 50 and 250 bp. For each sequence we see a gradual increase in primer cost as T_m_ is raised: suggesting MCMC-ODPR’s ability to effectively design reusable primers is constrained due to the rarity of suitable priming sites as T_m_ increases, Figure 2. The global coverage of sequences in our dataset by our designed primers was also calculated as a function of T_m_, Figure 3. Unsurprisingly, the coverage decreased as melting temperature was increased again due to the increasing constraint on suitable priming sites for the required T_m_.

**Figure 4:**
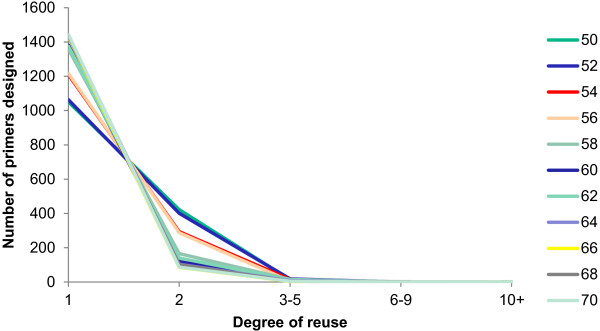
**Coverage of the designed primers over a Tm range.** The dataset of 247 FASTA sequences were input into MCMC-ODPR with no SNP processing and 10,000 optimization iterations. The degree of primer reuse generated MCMC-ODPR is plotted over a melting temperature range.

To observe the effect of a range of T_m_ on primer reuse, the number of primers for each level of redundancy at each T_m_ was plotted, Figure 4. It is clear that a higher degree of reusability is achievable at lower melting temperatures with the majority of reusable primers having 2x redundancy. Two primers that were reusable over 10 times were found at a T_m_ of 50 with one more found at a T_m_ of 56. We therefore suggest to users of MCMC-ODPR, that when possible, designing primers at slightly lower temperatures than ‘standard’ may make a large difference to cutting costs.

### Performance comparison of MCMC-ODPR with other primer design software

#### Comparison with other primer design software Primer 3 and PRIMER-BLAST

We compared MCMC-ODPR with primer design programs Primer3, Primer-BLAST, BatchPrimer3 and PAMPS. For primer design from single input sequences MCMC-ODPR was compared to Primer3, Primer-BLAST, and for multiple input sequences BatchPrimer3 and PAMPS. T_m_ = 60 was used for the design of primers from MCMC-ODPR, with 10,000 iterations. All of the programs we compared with MCMC-ODPR produced many more primers than would be required to amplify the target regions, with many amplicon targets occurring in the same area. Consequently, the total primer cost was noted, but the outputs were processed with a script such that non-overlapping primer pairs were selected that represented the maximum coverage of the target gene sequences.

##### Primer3 and PRIMER-BLAST for single input sequences

The Primer3 algorithm has been implemented in a number of other primer prediction programs, including BatchPrimer3 and Primer-BLAST. Primer3 and Primer-BLAST may only process one FASTA sequence at a time, therefore, the three barley gene sequences were randomly selected from our list of 247 barley genes and input into all three programs. Comparisons were made on the basis of the cost of designed primers in nucleotides, and the sequence coverage obtained from using these primers (Table 3). For the comparison of both programs with MCMC-ODPR the default parameters on the website were used, with a range of T_m_ of 57 to 63 degrees centigrade (60 optimum) with no allowed difference in T_m_ and primer lengths of 18-27 nucleotides (20 optimum). Both programs allow the user to specify the number of primer sequences returned, therefore the same number of primers as designed by MCMC-ODPR was selected. The PCR product size input into Primer3, Primer-BLAST and MCMC-ODPR was a minimum of 50 and a maximum of 250 base pairs. For the specificity check that Primer-BLAST performs to eliminate unspecific primer pairs the *Hordeum vulgare* non-redundant database was chosen for BLAST searching.

**Table 3:** Performance of primer design software for single sequence input

	**Program**	**Size of gene** (**bp**)	**Number of primers in design**	**Cost** (**nucleotides**)	**Coverage** (**bp**)	**Percentage coverage**	**Cost per covered base** (**nucleotides**)
Dehydrin 9	Primer3	1000	4(18)	80	401	40.1	0.2
	Primer-BLAST	1000	6(12)	116	527	52.7	0.22
	MCMC-ODPR	1000	9(10)	197	958	95.8	0.21
Beta-amylase 1	Primer3	3733	10(66)	160	691	18.51	0.23
	Primer-BLAST	3733	14(36)	334	998	26.73	0.33
	MCMC-ODPR	3733	34(34)	682	3636	97.4	0.19
C-repeat binding factor 3 like protein	Primer3	1515	8(26)	160	487	32.15	0.33
	Primer-BLAST	1515	6(14)	120	473	31.22	0.25
	MCMC-ODPR	1515	14(14)	274	1500	99.01	0.18

MCMC was input with one sequence corresponding to one gene (first column) and run for 10,000 iterations. The cost per bases covered in nucleotides was used as a comparison of performance. MCMC-ODPR performs less optimally with one sequence, yet was able to outperform single sequence programs Primer3 and Primer-BLAST in all examples but one.

Total number of primer sites identified shown in parentheses.

Primer3 designed 9 to 33 primer pairs per input sequence, from which 4 to 10 primers were selected to provide optimal coverage without overlap, Table 3. MCMC-ODPR achieved a lower cost per unit coverage than Primer 3 in all sequences except Dehydrin 9, where Primer 3 achieved a value of 0.2 compared with MCMC-ODPR, which achieved a value of 0.21. Primer-BLAST designed the number of primers specified in each case (12, 36 and 14), from which an optimal subset 6 to 14 was selected. The resulting cost of primer for the coverage achieved was consistently lower in MCMC-ODPR. Furthermore the amount of target sequence that was covered by the PCR systems designed by MCMC-ODPR was over 95% in all cases, whereas the maximum coverage achieved by Primer3 and Primer-BLAST was 40.1% and 52.7%, respectively.

##### Comparison with multiple sequence input programs: BatchPrimer3 and PAMPS

The same underlying algorithm as Primer 3 was compared with MCMC-ODPR with multiple sequences using BatchPrimer3, and the comparison with *PAMPS* allowed us to compare the capability of MCMC-ODPR to design reusable degenerate primers in terms of cost. A subset of 184 sequences was used from our list of 247 genes because of a limitation in PAMPS, which is restricted to sequences of 2000 base pairs or less. All sequences over 2000 base pairs were therefore removed. This subset was used for all three programs. BatchPrimer3 was used with the same default values for melting temperature and product size as with Primer3. BatchPrimer3 allows the user to input the desired number of primers to be returned per sequence, therefore for this value we chose the highest number of primers returned for all sequences by MCMC-ODPR, which was 10 primers. The analysis was performed first with allowing no difference in T_m_ between primer pairs for the BatchPrimer3 algorithm, as no difference in T_m_ is permissible with MCMC-ODPR, and then allowing the default T_m_ difference of 10 degrees centigrade between pairs. When no T_m_ difference was input to Batchprimer3 the sequence coverage of BatchPrimer3 was 29.7% with the total sequence coverage of MCMC-ODPR at a T_m_ of 60 degrees centigrade being 77.4% (Table 4). The cost of the primers designed by Batchprimer3 was 15,427 nucleotides, approximately half the cost of 30,103 nucleotides yielded by MCMC-ODPR, however, the coverage yielded was just over one third of that achieved with MCMC-ODPR. MCMC-ODPR achieved a lower cost per unit coverage than BatchPrimer3 of 0.19 compared with 0.25. When a maximum difference of ten degrees centigrade was allowed between primers for BatchPrimer3 it achieved a sequence coverage of 39.6% at a cost of 13,462 nucleotides, yielding a cost per coverage of 0.16 (not shown). Despite the lower cost of BatchPrimer3 in this case, MCMC-ODPR still achieved a greater sequence coverage and summary cost per unit coverage value close to that of BatchPrimer3 with the added constraint that MCMC-ODPR only designed primers with no difference in T_m_.

**Table 4:** Performance of primer design software for multiple sequence input

**Program**	**Number of primers designed**	**Cost** (**nucleotides**)	**Coverage** (**bp**)	**Percentage coverage**	**Cost per covered base** (**nucleotides**)	**Execution time** (**minutes**)*
MCMC-ODPR	1521(1786)	30103	162196	77.37	0.19	637
BatchPrimer3	372(1153)	15427	62292	29.71	0.25	2.98
PAMPS	1205(13598)	25284	39240	18.72	0.64	5.19

MCMC-ODPR was run with 184 sequences for 10,000 iterations with the cost per bases covered and runtime was used as a comparison statistic for performance. MCMC_ODPR outperformed multiple sequence input programs BatchPrimer3 and PAMPS in terms of cost per bases covered, yet was clearly slower in terms of runtime.

Total number of primer sites identified shown in parentheses.

* MCMC-ODPR Execution was performed on a 2.26 GHz Intel^®^ Core^™^ 2 Duo MacBook running Mac OS X version 10.6.8 with 2 GB RAM. Execution time for BatchPrimer 3 was provided by the BatchPrimer3 web server (primer design server 1) found at http://probes.pw.usda.gov/batchprimer3/. Execution for PAMPS was performed on a 2.31 GHz AMD Phenomtm 8650 Triple-Core Processor PC running Windows XP with 3.5 GB of RAM.

PAMPS designs degenerate primers according to *ad*-*hoc* pairwise alignments. The output of PAMPS is a list of degenerate primers consisting of a range of T_m_ with no information on which sequence its primers have been generated from. A script was therefore written to match the primers generated by PAMPS to our sequence dataset, keeping the same amplicon length constraints as above and only choosing primers with T_m_ = 60 degrees centigrade.

PAMPS designed 1205 primers that gave maximal coverage, which was comparable to the number generated by MCMC-ODPR at 1521 primers. PAMPS achieved the lowest coverage of 18.72% of the input sequences with a cost of 25,284 nucleotides. MCMC-ODPR outperformed PAMPS in terms of cost per unit coverage, with this value being calculated to be 0.64 for PAMPS.

From the execution times given in Table 4 it is clear that MCMC-ODPR is by far the slowest of the three compared programs. From Table 5 we see a dramatic decrease in the execution time when the number of input sequences is reduced, which shows that the primer space is highly influential towards execution time. MCMC-ODPR has not been optimized for speed and we recommend that users who require fast primer design should consider alternative programs, such as the comparison programs used in this study.

**Table 5:** MCMC_ODPR execution time with different numbers of sequences input

**Number of input sequences**	**Execution time** (**minutes**)
184	637
100	343.34
30	228.9

Execution time was measured when 184, 100 and 30 sequences were input into the program. Execution was performed on a on a 2.26 GHz Intel^®^ Core^™^ 2 Duo MacBook running Mac OS X version 10.6.8 with 2 GB RAM.

## Conclusions

MCMC-ODPR is a useful addition to the suite of primer design programs available in particular reducing costs through improved solutions to the MDPSP. The bench tests carried out in this study show that MCMC-ODPR consistently produced the lowest cost primer design all cases but two, but even then the slightly higher cost achieved a much larger coverage. Consequently, MCMC-ODPR was the most economically efficient primer design in all cases. The comparisons with other software show that by combining degeneracy with optimal primer reuse the user may increase the coverage of the sequences obtained by the designed primers at significantly lower costs. MCMC-ODPR’s enumeration through an input temperature range will allow the user to observe the trade off between T_m_ and the suitability of the sequence space for invoking reuse. However, as MCMC-ODPR is an optimization technique that utilizes the Metropolis-Hastings algorithm for optimizing primer reuse, it is intrinsically slow when compared to heuristic algorithms, and may possibly require a number of hours for multiple sequences, especially if a range of T_m_ has been input.

## Availability and requirements

**Project name**: MCMC-ODPR

**Project home page** [**permissions have been granted for open** (**anonymous**) **access**]: http://www2.warwick.ac.uk/fac/sci/lifesci/research/archaeobotany/downloads/MCMC_ODPR

**Operating system**(**s**): MacOSX

**Programming language**: Perl, Java

**License**: GNU General Public License (GNU-GPL)

**Any restrictions to use by non**-**academics**: none.

## Competing interests

The authors declare that they have no competing interests.

## Authors’ contributions

JDM and JLK developed the program. JLK wrote the manuscript and implemented the program. SAP helped in design of the program. RGA designed and supervised the project. All authors tested the program, and read and approved the final manuscript.
